# Non-invasive Imaging in the Evaluation of Cardiac Shunts for Interventional Closure

**DOI:** 10.3389/fcvm.2021.651726

**Published:** 2021-06-18

**Authors:** Kuberan Pushparajah

**Affiliations:** ^1^School of Biomedical Engineering and Imaging Sciences, King's College London, St Thomas' Hospital, London, United Kingdom; ^2^Department of Paediatric Cardiology, Evelina London Children's Hospital, London, United Kingdom

**Keywords:** imaging, interventions, echocardiography, shunt, cardiac magnet resonance imaging, computed tomography, congenital heart disease, multimodality

## Abstract

Multimodality imaging provides important information to guide patient selection and pre-procedural decision making for shunt lesions in CHD. While echocardiography, CT, and CMR are well-established, 3D printing and now virtual reality imaging are beginning to show promise.

## Introduction

Cardiac shunts can be defined as abnormal blood flow connections in place of or in addition to the normal circulation with potentially detrimental effects. Where indicated, these shunts can be closed by open surgical techniques or less invasive percutaneous transcatheter approaches. There is a wide array of transcatheter interventions in congenital heart disease (CHD) for the treatment of relatively simple shunt lesions such as an isolated atrial septal defect to the more complex adverse intracardiac and extracardiac shunts in a Fontan circulation. The variation and complexity of lesions are not limited to anatomy but include their hemodynamic consequences. Precise evaluation and patient selection are therefore key to determining a successful outcome.

Non-invasive imaging is the first-line investigation prior to invasive confirmatory or diagnostic evaluation at the time of catheter intervention. The latter includes invasive intracardiac echocardiography, biplane angiographic fluoroscopy, and rotational angiography. In this review, we will discuss the role of non-invasive imaging for planning interventional closure of cardiac shunts in CHD.

The key roles of imaging in interventional closure of shunts include (1) determining the threshold or need for intervention, (2) assessing suitability for intervention, (3) guidance of interventions, and (4) monitoring for complications, residual lesions, and remodeling. This review will focus on the first two points only.

## Threshold for Intervention

The threshold for catheter or surgical intervention is commonly determined by a combination of the type, size, and hemodynamic effect of the lesion combined with patient symptoms and exercise tolerance. There are several international expert consensus and guideline documents outlining current best practice in this area (1, 2). Imaging evaluation of hemodynamic effects includes size and direction of the shunt, estimation of pressure gradients, and volume loading effects on chamber enlargement and function.

In common with all left-to-right shunts, an intracardiac shunt is hemodynamically significant if there is evidence of chamber enlargement distal to the shunt and/or evidence of sustained Qp:Qs ≥ 1.5:1 (Qp = pulmonary blood flow, and Qs = systemic blood flow). An intracardiac shunt not meeting these criteria would be described as small or trivial (2). Percutaneous or surgical closure may be considered for adults with ASD when the net left-to-right shunt (Qp:Qs) is 1.5:1 or greater, PA systolic pressure is 50% or more of systemic arterial systolic pressure, and/or pulmonary vascular resistance is greater than one third of the systemic resistance (2). Right-to-left shunts in postoperative single-ventricle circulation are considered for closure where it causes significant systemic desaturation.

## Suitability for Intervention

Suitability for catheter intervention is predominantly based on the type and anatomy of the shunt. The former comprises the number of defects (e.g., multiple ASDs or VSDs), size, shape, and margins of the defect and identification of other associated lesions.

The types of shunts can largely be categorized into intracardiac shunts including atrial septal defects (ASD), ventricular septal defects (VSD), and atrioventricular septal defects (AVSD), and extracardiac shunts such as patent ductus arteriosus (PDA), coronary artery fistulae, aortopulmonary collaterals, venous collaterals, and pulmonary arteriovenous malformations. In practical terms, they can also be grouped into two categories summarized in Table 1.

**Table 1:** Summary of shunt lesions in congenital heart disease.

**Type of shunt**	**Anatomical defect**
1) Simple shunts	Atrial septal defect (ASD)
	Ventricular septal defect (VSD)
	Patent ductus arteriosus (PDA)
2) Complex shunts	
a) Pre-operative	Atrioventricular septal defect (AVSD) Sinus venosus atrial septal defect (SVASD) Coronary artery fistulae Pulmonary arteriovenous malformations
b) Post-operative	Single-ventricle surgical palliation: Superior cavopulmonary connection (Glenn shunt/hemi-Fontan) Fontan circulation • Fenestration/baffle leak • Systemic to pulmonary venous collaterals • Aortopulmonary collaterals • Pulmonary arteriovenous malformations
	Atrial switch for transposition of the great arteries: • Systemic or pulmonary venous baffle leak
	Previous ASD/VSD closure (isolated repair or component of complex CHD surgery) Residual ASD or VSD

## Non-invasive Imaging Options

A multimodality approach to imaging is well-established as outlined by several international consensus and guideline documents from the ESC/AHA/ASE (3, 4).

### Echocardiography

#### Two-Dimensional (2D) Echocardiography

Two-dimensional echocardiography is the key imaging modality for the evaluation of shunt lesions due to its wide availability in clinical practice and low cost. It is an effective first-line imaging and screening tool to define the anatomy and hemodynamic effects in shunt assessments (5). With skilled operators, multiple 2D views can be obtained to achieve a three-dimensional perception of a lesion using either transthoracic (TTE) or transesophageal echocardiography (TOE). This is easier for intracardiac shunt lesions but can be more challenging or indeed impossible in some extracardiac shunt lesions due to the available acoustic windows. Color Doppler and spectral Doppler (pulse-wave and continuous-wave Doppler) used in sequence allow for assessment of the presence, direction, and velocity of a shunt. The peak continuous-wave Doppler velocity across a shunt can be translated into a pressure gradient across two sides of a shunt lesion by applying the modified Bernoulli equation, hence providing important hemodynamic data.

In intracardiac left-to-right shunt lesions, the assessment of the size of the shunt can also be calculated by estimation of the ratio of pulmonary blood flow (Qp) to systemic flow (Qs). Estimation of flow can be achieved by pulsed-wave Doppler-derived velocity time integral (VTI) and calculated surface area across the right ventricular outflow tract (RVOT) and left ventricular outflow tract (LVOT).

- Qp = RVOT VTI × π × (RVOT diameter/2)^2^
- Qs = LVOT VTI × π × (LVOT diameter/2)^2^

However, this estimation requires assumptions which include a laminar blood flow velocity profile, circular cross-sectional area, and fixed area size. However, in CHD, the cross-sectional area of the vessel not only is of varying shapes but also changes over the course of the cardiac cycle, while flow is often turbulent and parabolic. Hence, echo-derived flows and estimation of shunts can be unreliable. Additionally, it is only potentially useful in simple and isolated intracardiac shunt lesions such as ASDs, VSDs, and PDA. The magnitude of shunting can also be indirectly estimated by assessment of atrial or ventricular dilation as a consequence of increased volume loading.

#### 3D Echocardiography

The application of 3D echocardiography is now more commonplace and is becoming an increasingly available imaging tool in routine clinical practice. There are published standards for image acquisition, post-processing, and display of 3D echocardiographic imaging in CHD (6). It is particularly useful for en-face 3D projections of septal walls for evaluation of septal defects, providing an improved appreciation of rims of a defect and its relationship to surrounding structures (7–9). Improvements in matrix array probes and software also allow for rapid image acquisition in a routine clinical workflow even in small babies and children with fast heart rates without the need for breath-holds (9).

### Cardiac MRI

Cardiac MRI (CMR) is widely used as a cross-sectional imaging tool that allows for 2D and 3D imaging of cardiac anatomy along with accurate and reproducible ventricular volumes and flows. The high accuracy, reproducibility, and absence of ionizing radiation make this an ideal imaging adjunct and gold standard for many forms of CHD (10). Quantification of intracardiac shunts (Qp:Qs) by 2D phase-contrast (PC) CMR has been demonstrated to be as accurate as invasive oximetric studies (11) and is now a routine gold-standard non-invasive imaging technique for the assessment of cardiac shunts. The assessment of shunts can either be measured directly in large vessels or be inferred from flow quantification across vessels in different parts of the circulation by phase-contrast (PC) CMR. In vessels where it is not possible to measure flow due to their small size or presence of turbulent flow due to the limitations inherent to PC CMR (12), it is possible to accurately calculate this flow from measurements in other parts of the circulation (13, 14). Four-dimensional flow (4D flow) is increasingly being used to measure flows in multiple sites of the circulation simultaneously and can replace 2D PC flow (15). It is particularly useful in complex lesions such as the Fontan circulation and eliminates the need for specialist input to plan PC flows in complex geometries at the time of acquisition. However, it requires longer scan duration and additional image post-processing tools.

Ventricular stroke volumes derived from volumetry can be used to assess Qp and Qs, but these can be less accurate than PC flow assessment, particularly in the right ventricle where ventricular segmentation can be more challenging and affect the accuracy of stroke volume quantification (16). Ventricular function and volumes further quantify the hemodynamic consequences of cardiac shunts. Data from CMR also gives indirect information on the pulmonary vascular resistance, and studies have indicated that a baseline Qp:Qs ≤ 2.75 in biventricular circulations with left-to-right shunts predicted a PVR ≥6 WU.m^2^ with 100% sensitivity and 48% specificity (17).

Anatomical details can be obtained from static or dynamic 2D bright blood imaging (static or cine SSFP) from operator-selected imaging planes of the region of interest not limited by acoustic windows such as in echocardiography. Detailed 3D whole-heart imaging can be obtained with and without the administration of intravenous gadolinium-based contrast agents. Non-contrast-enhanced imaging is achieved by 3D whole-heart ECG-triggered and respiratory navigated images (3DSSFP) (18). In these sequences, the respiratory navigator is set to only acquire images at end expiration and the ECG set to acquire in the systolic or diastolic rest periods, thus building a static 3D image of the heart. Contrast-enhanced 3D CMR angiography is not ECG gated but allows for multiple acquisitions over time, generating a time-resolved 3D angiogram (19). The 3D images can be used to help with procedural planning including site for vascular access to improve access to a lesion and definition of the optimal fluoroscopic imaging planes during cardiac catheter interventions (20) and produce 3D-printed heart models where needed (18). While CMR can generate dimensions and rims of defects such as muscular VSDs, it is less accurate for thinner structures such as ASDs and membranous portions of the VSD due to the spatial resolution compared to echocardiography and CT. A standard CMR protocol for CHD would include assessment of PC flows, ventriculography from cine SSFP stacks, and some form of 3D whole-heart imaging as a minimum dataset. Detailed considerations for specific lesions are outlined in consensus documents (21).

### Cardiac CT

Cardiac CT is an increasingly popular cross-sectional imaging modality due to its low-radiation dose exposure, easy access, short examination time, and high image resolution (19, 22). The short scanning time reduces the need for breath-holds and general anesthesia, thus clinically useful for unstable patients who can only tolerate short examinations or un-cooperative patients who may be able to lie still for very short periods of time.

ECG gating minimizes the effect of cardiac motion by acquiring at a fixed point in the cardiac cycle. In retrospective ECG gating, images are acquired continuously throughout the cardiac cycle and the operator can retrospectively reconstruct images with least motion artifacts, thus producing the best images but increased radiation exposure. This is particularly used for small structures such as coronary arteries which are prone to motion artifact (19). In prospective ECG triggering, images are obtained at a set time point, often during mid-systole or mid-diastole when the heart is assumed to have the least motion. Radiation dose is thus reduced, with the risk of repeat scanning in the event of arrhythmia or incorrect phase selection. Non-ECG-gated images can also be acquired generating the least radiation, but suspectable to the most motion artifact. Breath-holds and lowering of the heart rate with medication can help further reduce motion. Imaging the Fontan by CT is more challenging, and several modified approaches are proposed to enhance venous phase imaging of the pulmonary arteries (23).

Similar to CMR, CT provides comprehensive information of cardiac anatomy and surrounding vasculature in a three-dimensional view, with a higher level of detail but requiring the administration of intravenous contrast. The latest generation of multidetector scanners is competitive, producing a spatial resolution of <0.5 mm (0.8–1.5 mm for CMR) and temporal resolution of <50 ms (24). Examples of this include confirmation of pulmonary venous drainage in SVASD and PDA anatomy for intervention. Additionally, the high spatial resolution provides better assessment of ASD and VSD defect size and rims compared to CMR.

### 3D Printing

3D printing has been used for a variety of clinical applications in CHD which extend to planning for catheter interventions (25, 26). This typically uses 3D CT and CMR data, although 3D echocardiography is increasingly being utilized (27), which is very beneficial for ASDs and atrioventricular valves which are less well-seen on CT or CMR. This is mainly due to the ease of segmentation with CT and CMR data compared to 3D echocardiographic data which suffers from an inferior tissue-to-blood pool contrast. Three-dimensional (3D) data in DICOM (Digital Imaging and Communications in Medicine) format is loaded into 3D visualization software, segmented by an expert via a variety of segmentation tools (28) then exported into stereolithography (stl) file into 3D visualization software and transformed into computer-aided design format that can be converted into a physical object using a 3D printer. The stl files themselves can be interrogated in a 3D PDF reader without the need for printing in some cases where the user only needs to understand spatial relationships. The measurements in 3D models are also valid. In a large multicenter study of 40 patients with complex CHD, 3D-printed models were found to be highly accurate with mean differences of 0.27 ± 0.73 mm between measurements performed on the 3D-printed models and source CT/CMR images (29).

3D printing is used to produce simulations of catheter occlusions of ASDs, VSDs, and coronary artery fistulae (30, 31). With appropriate selection of printing material, these models are radiopaque and suitable for fluoroscopic bench-testing. This allows for better patient selection and appropriate selection of devices ahead of the procedure (32). Although PDA device occlusion is largely performed based on pre-procedural 2D echocardiography data alone or CT and CMR in some cases, 3D printing has been used for planning these interventions, allowing interventionalists to simulate and practice device deployment within the models themselves, thereby decreasing fluoroscopic and procedural times (33). In a surgical planning study, 3D visualization from static 3D printed models was found to improve understanding of anatomy and helped redefine the surgical approach in up to 47% of cases with complex congenital heart disease with significant modifications in 25% of cases (29). Prospective studies in catheter interventional planning for CHD may have potential to identify similar benefits.

### Virtual Reality

Virtual reality (VR) is inexpensive, reproducible, and importantly, swiftly available at the point of care compared to 3D-printed models (34). Advances in computerization and software development mean that it is now feasible to project 3D echocardiographic images into a VR environment (35) similar to cardiac CT and CMR data (36). This is an important development as we are better able to capture the dynamic geometrical and dimensional changes of a defect throughout the cardiac cycle, compared to traditionally static 3D CT and CMR data.

The application of VR for interventional planning as 2D and 3D echocardiographic data for catheter and device selection requires multiple measurements in different imaging planes. Linear measurements made in this way can be difficult to translate particularly in complex lesions which can introduce errors in perception and communication between the imager and the interventionalist. Volume-rendered 3D echocardiographic images of patients with ASDs and VSDs using custom codes have been integrated with models of devices in a VR environment for simulation of device selection in planning catheter interventions to closure intracardiac shunts (37). Additionally, it has been applied to patient education leading to a reduction in patient anxiety and procedure time for PFO and ASD closure compared to a control group (38).

Early benefits are already being seen in the VR application of surgical planning for atrioventricular valve surgery in CHD (39).

## Non-invasive Imaging for Specific Shunt Lesions

The application of the role of non-invasive imaging illustrated in specific lesions is described in the following section.

### Atrial Septal Defect (ASD)

In a large majority of cases, assessment of ASD is by echocardiographic imaging alone. Transesophageal echocardiography (TOE) is the best echo imaging tool due to the posterior location of the atria, and the superior acoustic windows available from TOE do define the number, size, location, and rims of a septal defect. Significant lesions with a left-to-right shunt result in right atrial and ventricular dilation. Assessment of pulmonary pressures can be inferred from indirect measures such as tricuspid regurgitant jet velocities and pulmonary end diastolic Dopplers. There are useful guides on standard 2D and 3D imaging for the assessment of ASDs (40, 41). The key aim is to define the location and rims of the defect(s) in relation to surrounding structures such as the SVC, IVC, aorta, right pulmonary veins, and mitral valve which would serve as a landing zone for the device rims, thus determining suitability and optimal device sizing.

**Figure 1:**
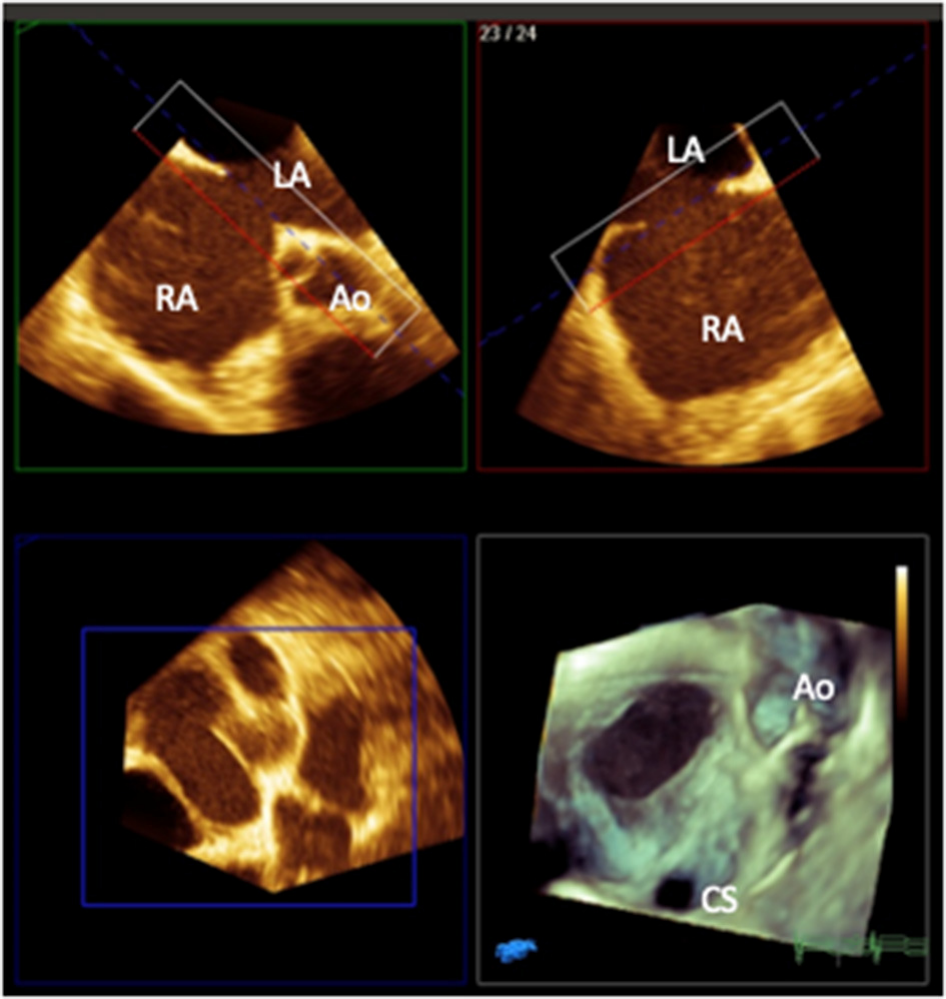
3D multiplanar reformat and rendered image (bottom right) of a transesophageal image of a secundum atrial septal defect with a deficient aortic rim as viewed from the right atrial aspect (Ao, aorta; CS, coronary sinus; RA, right atrium; LA, left atrium). Image courtesy of Alexandra Savis, Evelina London Children's Hospital.

Three-dimensional echocardiography can further enhance visualization of ASDs particularly in the setting of complex or multiple defects (7) (Figure 1). Where there is a concern about the presence of concurrent anomalous pulmonary venous drainage which may need surgical intervention instead, TOE can be helpful, but 3D imaging with CT or CMR is more definitive. In rare occasions, CT is required for coronary imaging in the presence of a retroaortic circumflex coronary artery which may preclude device closure of an ASD (42).

### Sinus Venosus ASD (SVASD)

**Figure 2:**
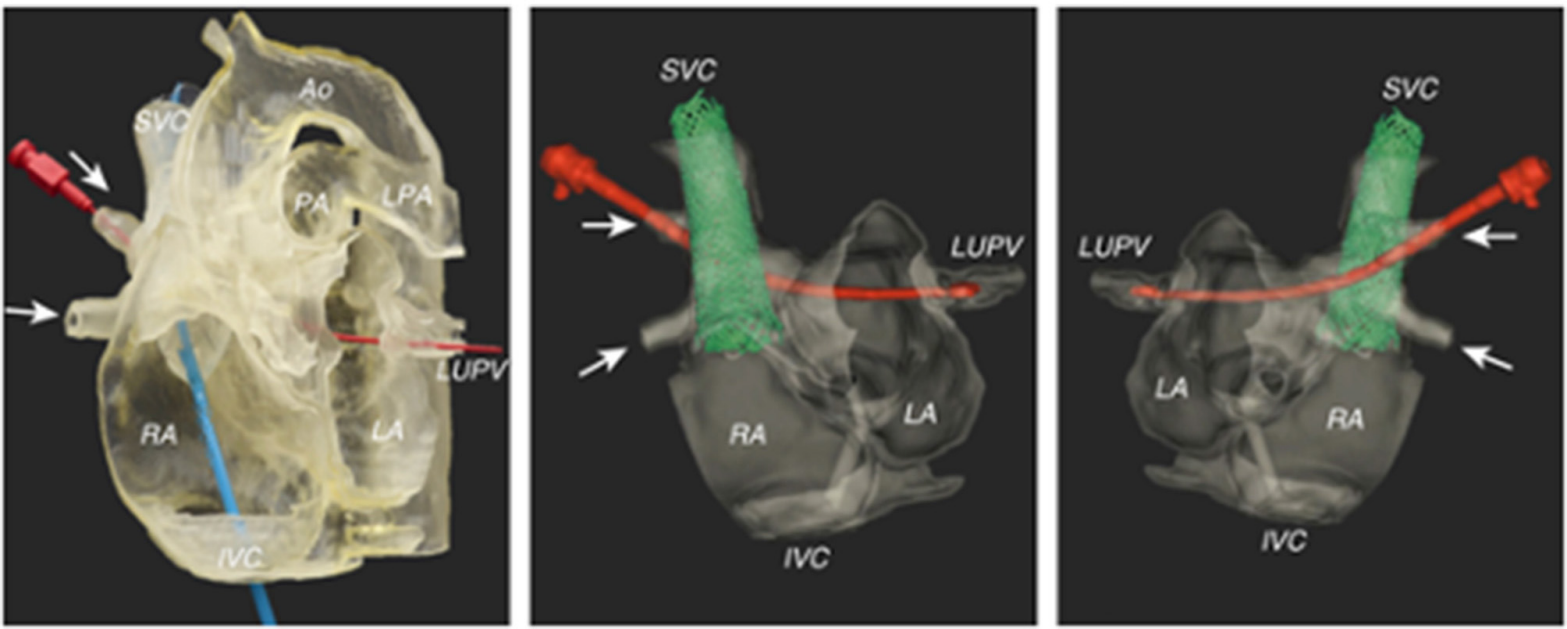
Pre-procedural imaging for sinus venosus ASD using a 3D-printed model for simulation of the stenting procedure. A red probe is inserted in the path of the right upper pulmonary venous drainage to the left atrium. Images reproduced with permission from Velasco Forte et al. (30).

Sinus venosus ASD is characterized by deficiency of the common wall between the superior vena cava (SVC) and the right upper pulmonary vein (RUPV), which is no longer committed to the left atrium. It constitutes up to 11% of all cases with ASD (43) and is used to be limited to surgical repair which can now be considered for catheter intervention with good results (44). The procedure involves implantation of a covered stent which replaces the deficient posterior wall of the SVC, thereby closing the SVASD and redirecting the anomalous pulmonary veins into the LA behind the stent. Use of 3D CT and CMR data rendered into 3D prints allows for case selection and pre-procedure planning (30), as not all cases are suitable for this procedure and some may still need to undergo surgery (Figure 2). In one recent study using TOE and peri-procedural fluoroscopic balloon interrogation, patients' exclusion of all defects caudally extending toward the oval fossa and right upper pulmonary veins draining beyond the cavoatrial junction resulted in successful outcomes for stent treatment of sinus venosus ASD (45).

### Ventricular Septal Defect (VSD)

**Figure 3:**
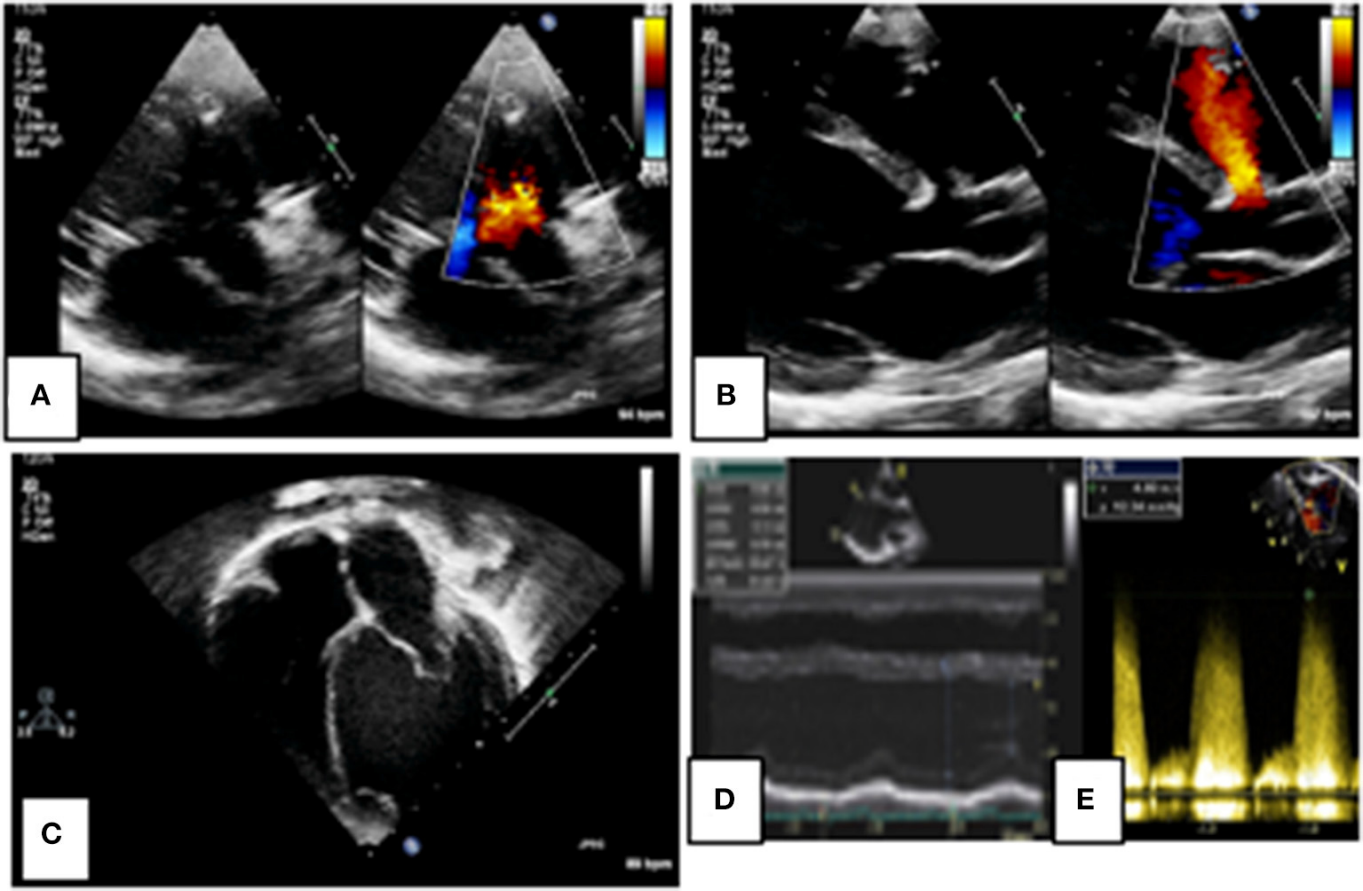
2D echocardiographic assessment of a peri-membranous ventricular septal defect (VSD). Figures **(A,B)** define the site and size of the lesion as a small peri-membranous VSD with a left-to-right shunt on color Doppler. There is evidence of left atrial and ventricular dilation in figure **(C)** indicating a significant left-to-right shunt, as measured on M-mode **(D)**. The shunt velocity is high on spectral Doppler indicating favorable hemodynamics for VSD closure **(E)**.

Ventricular septal defects are common (46) and in the setting of a significant left-to-right shunt can be amenable to transcatheter device closure. Determination of the size of the shunt is typically from assessment of left atrial and left ventricular dilation and pulmonary pressures from the Doppler velocity of VSD shunt (Figure 3). While multiple 2D views of the septum can help evaluate the position of a defect, it can be challenging to visualize the entirety of the VSD in a single plane and accurately measure its dimensions. 3D echocardiography provides an en-face projection of the defect(s) with all its rims and depth of view of relationships to surrounding structures. This allows for the evaluation of suitability for device closure incorporating irregular shapes, multiple defects, and evaluation of potential damage to the tricuspid and aortic valves.

### Patent Ductus Arteriosus (PDA)

**Figure 4:**
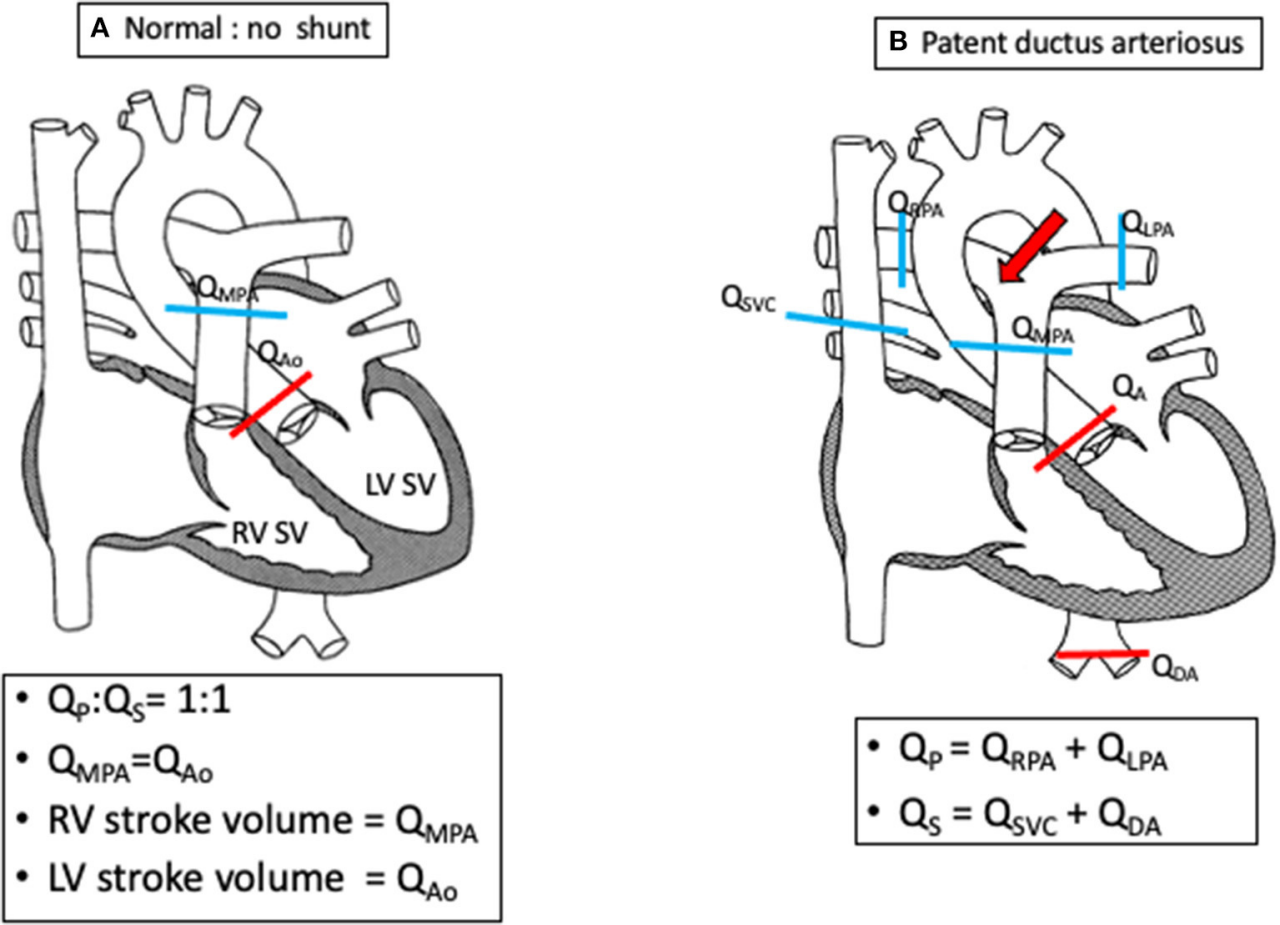
Schematics demonstrating shunt calculations (Qp:Qs) in a normal heart **(A)** and in a PDA **(B)** using flow quantification from cardiac magnetic resonance imaging.

**Figure 5:**
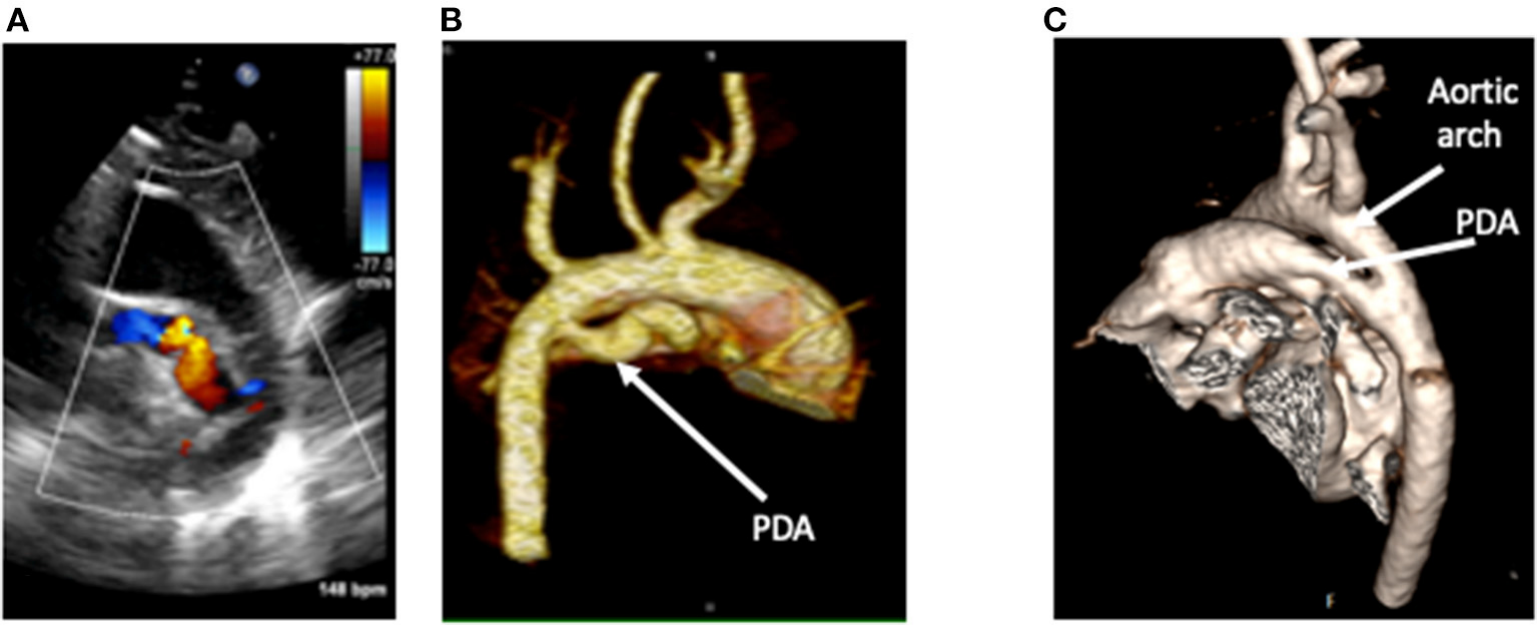
A tortuous duct as seen on 2D color Doppler echocardiography **(A)**, with clear delineation of the shape, dimensions, and insertion points on contrast-enhanced CT **(B)**. This is in comparison to a straight duct seen on CT in image **(C)**. CT Images courtesy of Dr. Sujeev Mathur, Evelina London Children's Hospital.

A patent ductus arteriosus (PDA) is a common source of a left-to-right shunt. The majority of imaging is done by 2D echocardiography which includes assessment of the size, shape, and hemodynamic consequences of the PDA. In some cases, accurate estimation of the PDA shunt is needed and can be accurately provided by CMR (Figure 4). It is particularly useful in the assessment of the PDA contribution in cases with simultaneous other sources of left-to-right shunt such as an ASD or VSD. As previously mentioned, contrast-enhanced CT and 3D CMR imaging can define the ductal anatomy in selected cases with complex ductal morphology (Figure 5), thus aiding decision making for the optimal approach for vascular access. It is also very useful for delineation of calcification which can pose a risk of vessel rupture during intervention such as calcified PDA in older patients (47).

### Coronary Artery Fistulae

**Figure 6:**
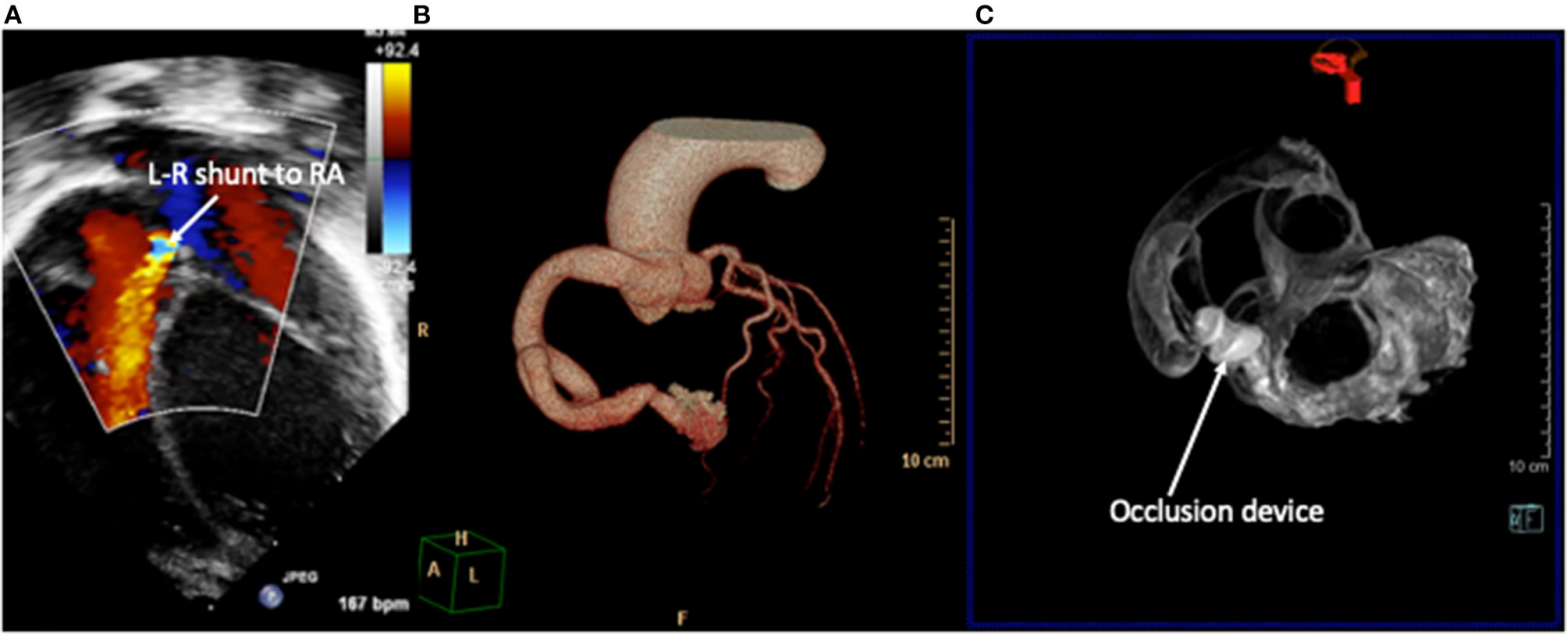
Right coronary artery fistula to right atrium (RA). **(A)** 2D echocardiography with color Doppler demonstrates the drainage point of the fistula into the RA at high velocity. Images courtesy of Israel Valverde, King's College London. The course of the fistula is better defined on contrast-enhanced CT **(B)** which allows for simulation of device occlusion as shown in a rotational CT image in image **(C)**.

Coronary artery fistulae or coronary cameral fistulae are an abnormality of coronary artery termination into a chamber of the heart. This can involve any coronary artery and exit sites into any cardiac chamber and have multiple feeding vessels and multiple drainage sites (48). Symptom severity depends on the degree of shunt and left- or right-heart overload. Two-dimensional echocardiography is an effective screening tool, and the origin and exit are more easily seen. The course of the defect, bends, turns, and areas of narrowing are more difficult, but dimensions of fistula can be estimated on 2D imaging. Functional assessment includes volume load, function, and estimation of the severity of the shunt. Of all (21/24), 87.5% were correctly and accurately diagnosed by echocardiography (49). The interventionalist will aim to occlude the fistula artery as distally and as close to its termination point as possible, so as to avoid any possibility of occluding branches to the normal myocardium. Where these patients previously needed invasive coronary angiography to provide detailed diagnosis, cardiac CT is the preferred imaging modality and can be used to produce 3D models to the evaluation of suitability for interventions (31) (Figure 6) to help define the site, size, and potential landing zone for the occlusion device(s) without risk of embolization or occlusion of important coronary connections.

### Intracardiac Shunts Post Atrial Switch

Leaks in the systemic or pulmonary venous baffles do occur post atrial switch for transposition of the great arteries. This can result in either pulmonary overcirculation or systemic desaturation and is amendable to catheter intervention to occlude the resulting shunt (50, 51). These can be difficult to identify from standard echocardiography, and an agitated saline contrast study is advised where there is a clinical suspicion. In one study, agitated saline contrast studied identified a baffle leak in up to 65% of patients compared to standard imaging (52). Cardiac MRI is also able to assess for baffle leaks employing cine SSFP imaging of the superior and inferior vena cava pathway long-axis views with oblique coronal planes parallel to the vena cava baffles combined with measurements of Qp:Qs from PC imaging (53).

### Fontan

Single-ventricle surgical palliation in the form of a Fontan circulation can result in multiple sources of shunt. This includes right-to-left shunts across a fenestration within the inferior limb (lateral tunnel or extracardiac conduit), lateral tunnel baffle leak, systemic to pulmonary venous collaterals, and intrapulmonary arteriovenous malformations. Left-to-right shunts are a consequence of aortopulmonary collaterals. These sources of shunt can be detrimental to the patient and addressed by transcatheter occlusion (54). Identification and quantification of these shunts can be challenging by echocardiography, and CT is very challenging in a Fontan circulation due to the passive flow and inherent delays in systemic venous return particularly in the inferior limbs of the circulation (55). Agitated saline contrast echocardiograms can help screen for the presence of significant right-to-left shunts. Contrast-enhanced CMR or 3D whole heart can further define the vessels of interest as targets for shunt occlusion.

**Figure 7:**
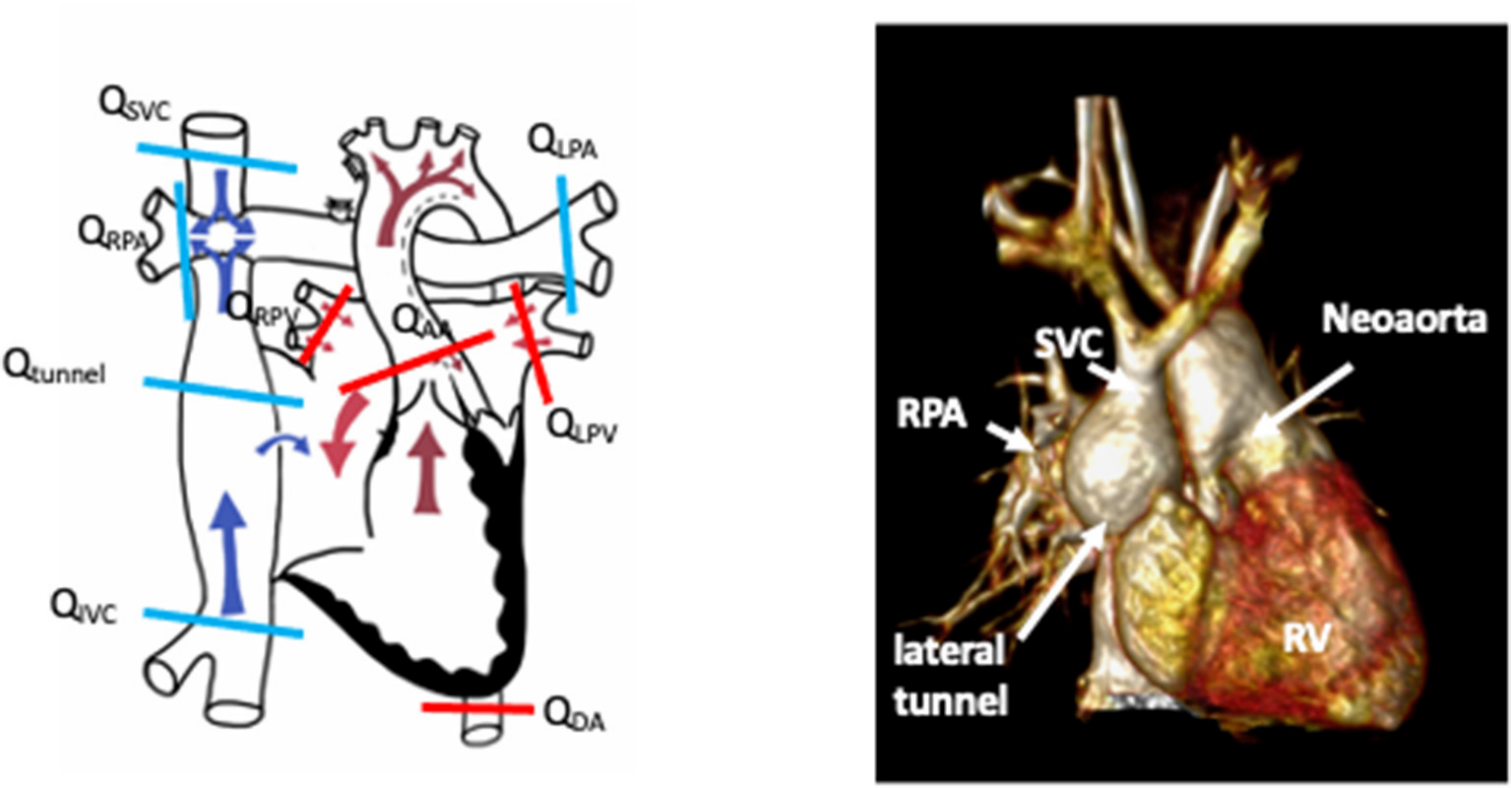
Schematic demonstrating sites of flow measurement in a lateral tunnel Fontan circulation by phase contrast CMR to facilitate shunt calculations. Schematic drawing courtesy of Dr. Aaron Bell, Evelina London Children's Hospital. The accompanying figure is a rendered 3D whole-heart image of a patient with a Fontan circulation for hypoplastic left heart syndrome. SVC, superior vena cava; RPA, right pulmonary artery; RV, systemic right ventricle.

Fenestration flow can be accurately measured by CMR and is driven by a balance between pulmonary vascular resistance and early diastolic ventricular function (56). Similarly, aortopulmonary collateral flow estimation can also be reliably assessed and quantified by CMR (13). The quantification of these shunts can be accurately calculated from PC-CMR flow measurements within other parts of the circulation (13, 14, 56), as detailed below and illustrated in Figure 7.

- Fenestration or baffle leak: (Q_SVC_ + Q_IVC_) – (Q_LPA_ + Q_RPA_).
- Aortopulmonary collaterals: Q_AAO_ – (Q_SVC_ + Q_IVC_) or (Q_LPA_ + Q_RPA_) – (Q_LPV_ + Q_RPV_).
- Qp:Qs ratio: (Q_LPV_ + Q_RPV_)/(Q_SVC_ + Q_IVC_).

## Summary

Multimodality imaging provides important information to guide patient selection and pre-procedural decision making for shunt lesions in CHD. While echocardiography, CT, and CMR are well-established, 3D printing and now virtual reality imaging are beginning to show promise.

## Author Contributions

The author confirms being the sole contributor of this work and has approved it for publication.

## Conflict of Interest

The author declares that the research was conducted in the absence of any commercial or financial relationships that could be construed as a potential conflict of interest.
